# Relationships between borders, management agencies, and the likelihood of watershed impairment

**DOI:** 10.1371/journal.pone.0204149

**Published:** 2018-09-20

**Authors:** Josh Epperly, Andrew Witt, Jeffrey Haight, Susan Washko, Trisha B. Atwood, Janice Brahney, Soren Brothers, Edd Hammill

**Affiliations:** Department of Watershed Sciences, Utah State University, Logan, UT, United States of America; Universidade de Brasilia, BRAZIL

## Abstract

In the United States, the Clean Water Act (CWA) establishes water quality standards important for maintaining healthy freshwater ecosystems. Within the CWA framework, states define their own water quality criteria, leading to a potential fragmentation of standards between states. This fragmentation can influence the management of shared water resources and produce spillover effects of pollutants crossing state lines and other political boundaries. We used numerical simulations to test the null prediction of no difference in impairment between watersheds that cross political boundaries (i.e. state lines, national or coastal borders, hereafter termed “transboundary”) and watersheds that cross no boundaries (hereafter “internal”). We found that transboundary watersheds are more likely to be impaired than internal watersheds. Further, we examined possible causes for this relationship based on both geographic and sociopolitical drivers. Though geographic variables such as human-modified land cover and the amount of upstream catchment area are associated with watershed impairment, the number and type of agencies managing land within a watershed better explained the different impairment levels between transboundary and internal watersheds. Watersheds primarily consisting of public lands are less impaired than watersheds consisting of private lands. Similarly, watersheds primarily managed by federal agencies are less impaired than state-managed watersheds. Our results highlight the importance of considering Integrated Watershed Management strategies for water resources within a fragmented policy framework.

## Introduction

Healthy freshwater systems are crucial for supporting diverse aquatic communities[1], the ecosystem functions performed by these communities[2,3], and the ecological services they provide[4–6]. The Clean Water Act of 1972 is the cornerstone of water management in the United States, serving as a benchmark by which the health of freshwater systems is assessed. The Clean Water Act emphasizes the authority of jurisdictions by allowing states to set their own water quality standards through the Total Maximum Daily Load program[7]. However, one potential issue with the Clean Water Act framework are watersheds that cross state boundaries, as these watersheds are technically subject to more than one set of water quality standards. Within water policy literature, Integrated Watershed Management (IWM) has been offered as an alternative to the jurisdictional fragmentation of state-defined standards. Based on John Wesley Powell’s 1878 vision of “watershed commonwealths,” IWM proposes that water resources should be managed at the watershed scale rather than adhering to man-made political boundaries[8]. Proponents of IWM argue that this rescaling of management would result in the most optimal social and ecological outcomes, as the grouping of stakeholders would mirror the spatial dimensions of their shared water resources[9].

IWM has been successfully applied to improve water conservation and public welfare in watersheds as geographically diverse as Ethiopia[10], India[11] and Alberta, Canada[12]. Despite this, there remain many technological and political hurdles to the widespread implementation of IWM[13,14], and its efficacy has only been presented on a case-by-case basis[10,11,13]. If we are to view IWM concepts as improvements to the Clean Water Act framework, we must first establish whether jurisdictional fragmentation is harming the integrity of United States watersheds. We aim to test this by determining whether United States watersheds that are intersected by state lines, national boundaries, or border a coastline (hereafter referred to as ‘transboundary’) are proportionally more impaired than watersheds that are contained within the bounds of a single state (hereafter ‘internal’).

There are compelling reasons to suspect that transboundary watersheds are proportionally more impaired than their internal counterparts. Transboundary watersheds may be more impaired due to state-by-state variability in water management. Not only do states use different indices to measure and regulate pollutants[15], but a single river system flowing through multiple states may be managed for different purposes [8,16,17]. Further, the priorities guiding water management—such as promoting valuable resources or supporting agricultural and municipal needs—can affect the water quality standards defined by each state[17]. The complexities inherent to interstate watershed management are also likely to apply to the management of coastal watersheds. In coastal watersheds, inland and marine water resources are often managed by multiple agencies with varying degrees of jurisdictional fragmentation. For example, approximately 41% of waters within U.S. maritime boundaries are established as Marine Protected Areas (MPAs) and overseen by an assortment of federal, state and territorial agencies. In addition, multiple states and even countries may affect the quality of coastal waters, therefore acting in a similar manner to a large lake that crosses state lines. For example, the Deepwater Horizon accident was centered off the coast of Lousianna, but affected the water quality of at least four states[18]. There has been a growing recognition that MPA, ocean, and coastal watershed managers will need to adapt more collaborative approaches to protecting their water resources against the stressors of pollution, overexploitation and climate change[19–21].

The effects of state and coastal variability in watershed management may also be exacerbated by the difficulties that come with dividing duties among managers. Due to their geographic locations, transboundary watersheds are inherently managed by more agencies than internal watersheds. Many have observed that as the number of agencies sharing a natural resource increases, so does the potential for conflict, miscommunication, and human error[22,23]. For example, under the Colorado River Compact of 1922, the Colorado River has been jointly managed by the seven western states within its drainage basin. Management problems have included overestimating the annual acre-feet the river can supply, prioritizing the water rights of some states and users over others, and disagreement over who is responsible for providing Mexico its guaranteed 1.5 million acre-feet per annum[24–26]. Conservationists have argued that directing efforts and resources towards these conflicts has come at the expense of solving environmental issues within the basin[27].

While these sociopolitical realities provide a basis for our research question, we must also consider potential mechanisms of transboundary watershed impairment from the surrounding landscape. First, spillover effects may be one driver of transboundary watershed impairment. There is evidence that industrial facilities in United States border counties discharge significantly higher volumes of air and water emissions than facilities in non-border counties, and that such activities may be incentivized by neighboring states sharing a portion of the environmental, monetary and human health costs of pollution[28,29]. Second, waterways can function both as hubs of human settlement and as borders between states. Major, state-splitting waterways flowing through dense population centers are not only subjected to stressors such as pollution and impoundment, but are also affected by the aforementioned difficulties of interstate watershed management[30–32]. In this way, transboundary watersheds may be uniquely vulnerable to the additive or synergistic effects of population centers and interstate management, while internal watersheds do not have this combination of factors. Third, waterways that act as borders are usually large (e.g. the Mississippi River); consequently, their upstream catchment areas are likely to be large as well. Expanding the area over which human impacts can occur may heighten the potential for pollution to be transported downstream.

A further geographic mechanism of watershed impairment is anthropogenic modification of the landscape, whether it be urban or agricultural development. There has been a wealth of literature on how landscape modification degrades watershed integrity[30,31,33–39]. Researchers have identified urban and agricultural land use thresholds that precipitate rapid losses of biotic integrity and increases in watershed impairment[40–42]. Although the impacts of these land uses can be abated by collaborative, watershed-wide policy solutions such as stormwater regulation and Beneficial Management Practices[43,44], the successful implementation of such policies is largely dependent on coordination between the relevant institutions and stakeholders. We suspect this level of harmonized management is more difficult to achieve in watersheds intersected by jurisdictional borders.

Balancing freshwater management for both human use and ecological health is made more complicated by having many stakeholders at the table, all of whom may have differing needs and ideological frameworks. Understanding the spatial trends of watershed impairment will be crucial for maintaining this balancing act and for enhancing cooperation among stakeholders. Here, we assessed whether the degree of jurisdictional fragmentation is a predictor of watershed impairment by investigating whether the likelihood of impairment is similar for transboundary and internal watersheds. To better understand the potential mechanisms behind our observed differences in transboundary and internal watershed impairment, we investigated socio-political (i.e. private land and the number of agencies managing land within the watershed) and geographical (i.e. modified land cover, upstream catchment area) drivers.

## Methods

**Fig 1:**
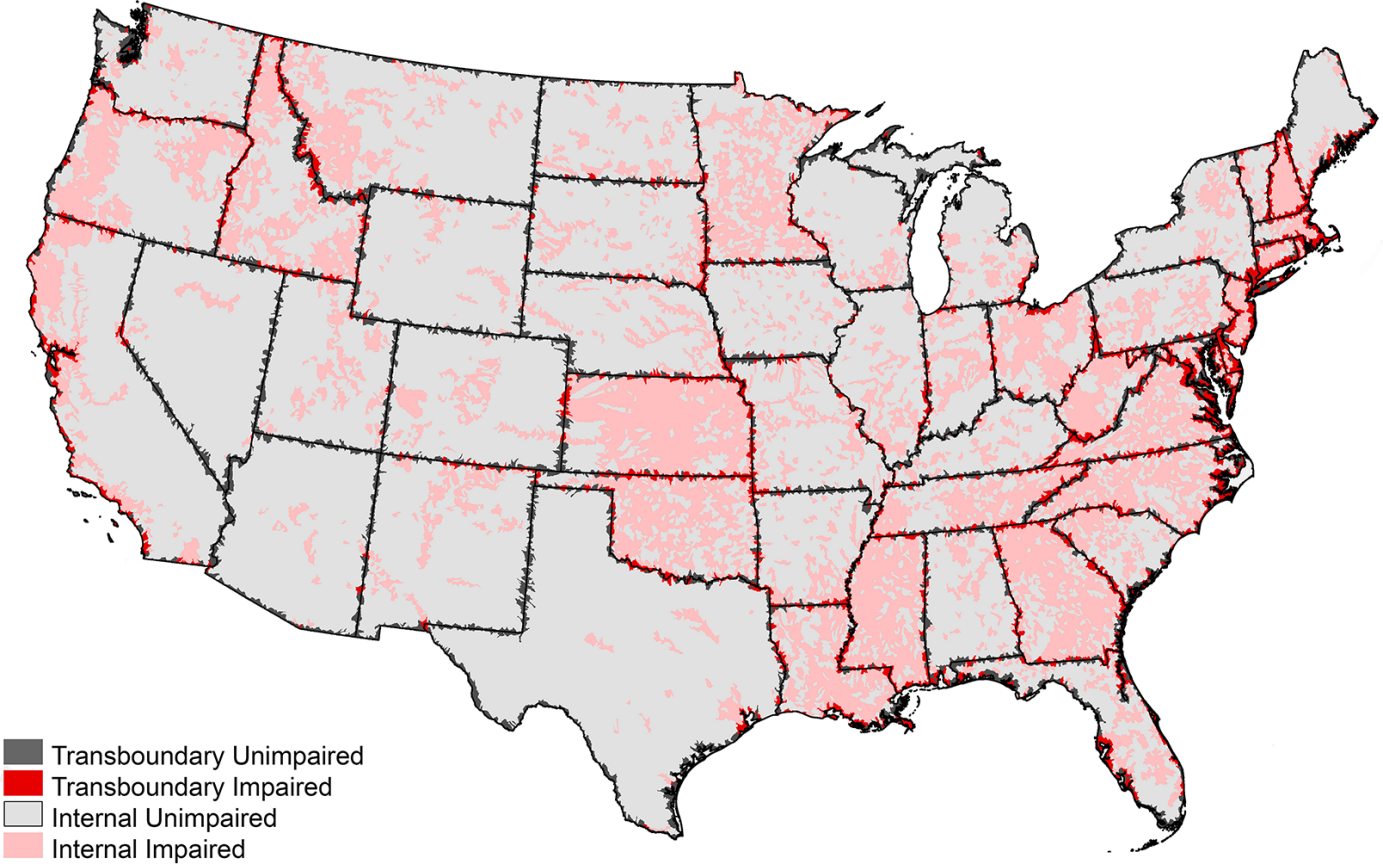
Impaired watersheds in the contiguous United States. Impairment is based on EPA’s 303(d) listing. Transboundary and internal watersheds are shown as impaired or unimpaired.

We identified watersheds across the contiguous United States using HUC 12 catchments from the United States Geological Survey’s Watershed Boundaries spatial dataset[45]. Each of the 60,726 watersheds was categorized as either transboundary or internal. Transboundary watersheds were defined as those crossing state or national borders or fringing a coastline (n = 6768), as listed in the Global Map Boundaries of the United States dataset[46]. Internal watersheds were defined as non-coastal watersheds that did not cross any borders (n = 53,958)[46]. Watershed impairment status was obtained through spatial data on 303(d) impairment violations from the United States Environmental Protection Agency (EPA) [47]. The Clean Water Act’s 303(d) list of impaired waterbodies provided information on impaired waters in each state, which was determined by whether the chemical, physical and biological characteristics of a waterbody meet state standards[47]. 303(d) impairment listings were joined to each HUC 12 catchment, creating a single, spatially explicit dataset comprised of impairment status and watershed group (i.e. transboundary or internal) (Fig 1). Based on this compilation, 35.8% of transboundary watersheds were listed as impaired (n = 2,424), while 27.0% of internal watersheds were listed as impaired (n = 14,560). Though larger catchment basins may be more representative of the larger stream networks found throughout the United States, the use of tributary-sized HUC 12 watersheds explicitly acknowledges the effects of pollution on small streams and tributaries that may be lost if aggregated to larger-scale watersheds.

### Transboundary vs. internal watershed impairment

Null models (bootstrapping) were developed in R v3.3.3 statistical programming language to compare the proportion of 303(d) impaired watersheds to total watersheds for both the transboundary and internal watershed groups[48]. We initially calculated a baseline by determining the proportion of watersheds classified as impaired within the full dataset of 60,726 watersheds. This baseline functioned as our null model, with the hypothesis that there was no difference between the proportion of impaired transboundary and internal watersheds. We then used numerical simulations to produce an estimate and distribution of proportional impairment for transboundary and internal watersheds, which was then compared to the null model. For each iteration of these simulations, we randomly sampled half of transboundary or internal watersheds, and the proportion of this sample that classified as impaired was calculated. This process was repeated for 1,000 iterations for both the transboundary and internal watershed groups, and these 1,000 iterations were used to calculate medians and 95% confidence intervals of proportional impairment for each watershed type. For each group, if the 95% confidence interval of the proportion impaired did not overlap the value estimated by the null model, then we deemed the group as having a significantly different proportion of impaired watersheds than expected. Our use of Null models provided information about the directionality of differences (i.e. whether a group has a greater or lesser proportion of impaired watersheds that would be expected). A Chi-square test was also used to corroborate that the difference in proportion of impaired internal or transboundary watersheds was significant.

### Relationships between watershed features and impairment

**Fig 2:**
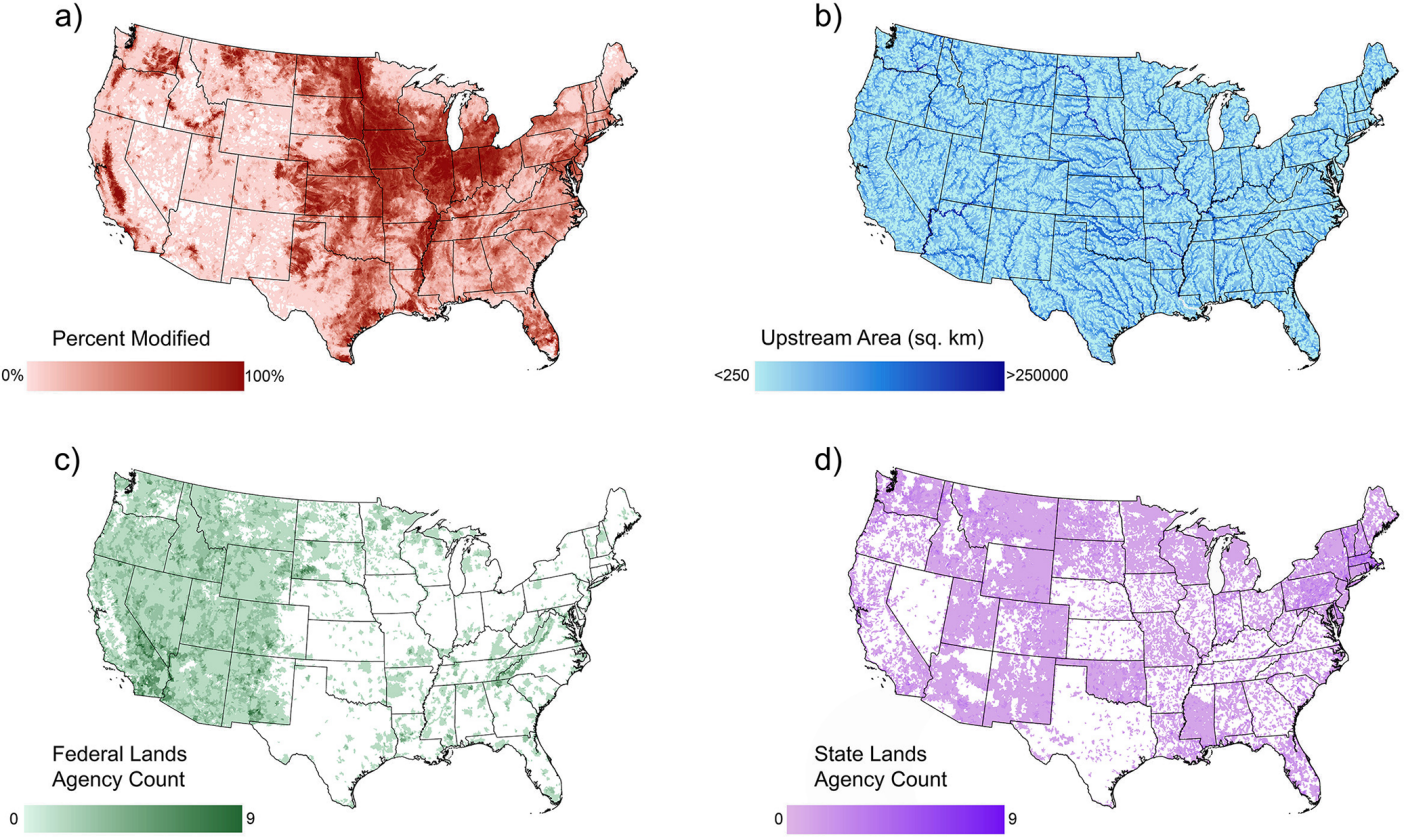
Physical and socio-political features assessed in this study. (a) Percentage of human modified land cover for all contiguous HUC 12 watershed. (b) Catchment sizes measured as amount of upstream area for all contiguous HUC 12 watershed. (c) The number of federal agencies working within each contiguous HUC 12 watershed. (d) The number of state agencies working within each contiguous HUC 12 watershed.

To investigate the potential for geographical differences to drive watershed impairment, we compared watershed catchment area and level of human-modified land cover with EPA impairment. We chose land cover as a proxy indicator of anthropogenic impacts to watersheds. Additional factors potentially leading to watershed impairment (i.e. impoundments, water abstraction, point-source pollution) were not included in this study due to a lack of data availability at a suitable scale. We used the National Land Cover Database (NLCD) to measure percentage of land modification as the aggregation of all NLCD classifications for agricultural and developed land cover (Fig 2A)[49]. Agricultural lands consisted of pasture, hay, and cultivated crops, while developed lands included open space and urban development of low, medium, or high density. Information on upstream catchment area was sourced from the HydroBASINS database and spatially joined to each HUC 12 watershed (Fig 2B)[50]. The relationships between land modification, upstream area, internal-transboundary status, and watershed impairment were tested using logistic regression models in R v3.3.3[48]. We specifically investigated how upstream catchment area and level of land modification affected the likelihood of impairment, and if these factors differed between transboundary and internal watersheds.

To understand the relationship between impairment and sociopolitical attributes of watersheds, we developed further tests to determine if watershed impairment correlated with a jurisdictional count. We sourced federal land management units from the Federal and Indian Lands datasets of the United States Geological Survey’s (USGS) National Map program[46]. These datasets designate the primary agency responsible for administering each federal land unit, as well as a secondary and tertiary agency of jointly-managed units (S1 Table, S2 Table). 1,769 of the 2,783 (63.6%) federal land units in the contiguous 48 states were listed as having only one administering agency, while 961 had two agencies and 53 had three agencies. State-owned land units and the sole agencies responsible for their management were delineated using areas listed in the Protected Areas Database of the USGS Gap Analysis Project[51]. This dataset only assigns a single local management agency for each state-owned public land unit. Each watershed was then spatially joined with the federal and state land units, producing counts of the unique federal and state management agencies, the sum of which we defined as the ‘jurisdictional count’ (Fig 2C and 2D).

Once the jurisdictional count was established, we tested the relationship between the jurisdictional count and watershed impairment using logistic regressions. We first tested whether watersheds with any amount of public lands (i.e. “public” watersheds) were more likely to be impaired than those without any public lands (i.e. “private” watersheds). Next, we specifically focused on public watersheds, and assessed the relationships between watershed impairment, the number of federal and state land management agencies, and transboundary status.

## Results and discussion

**Fig 3:**
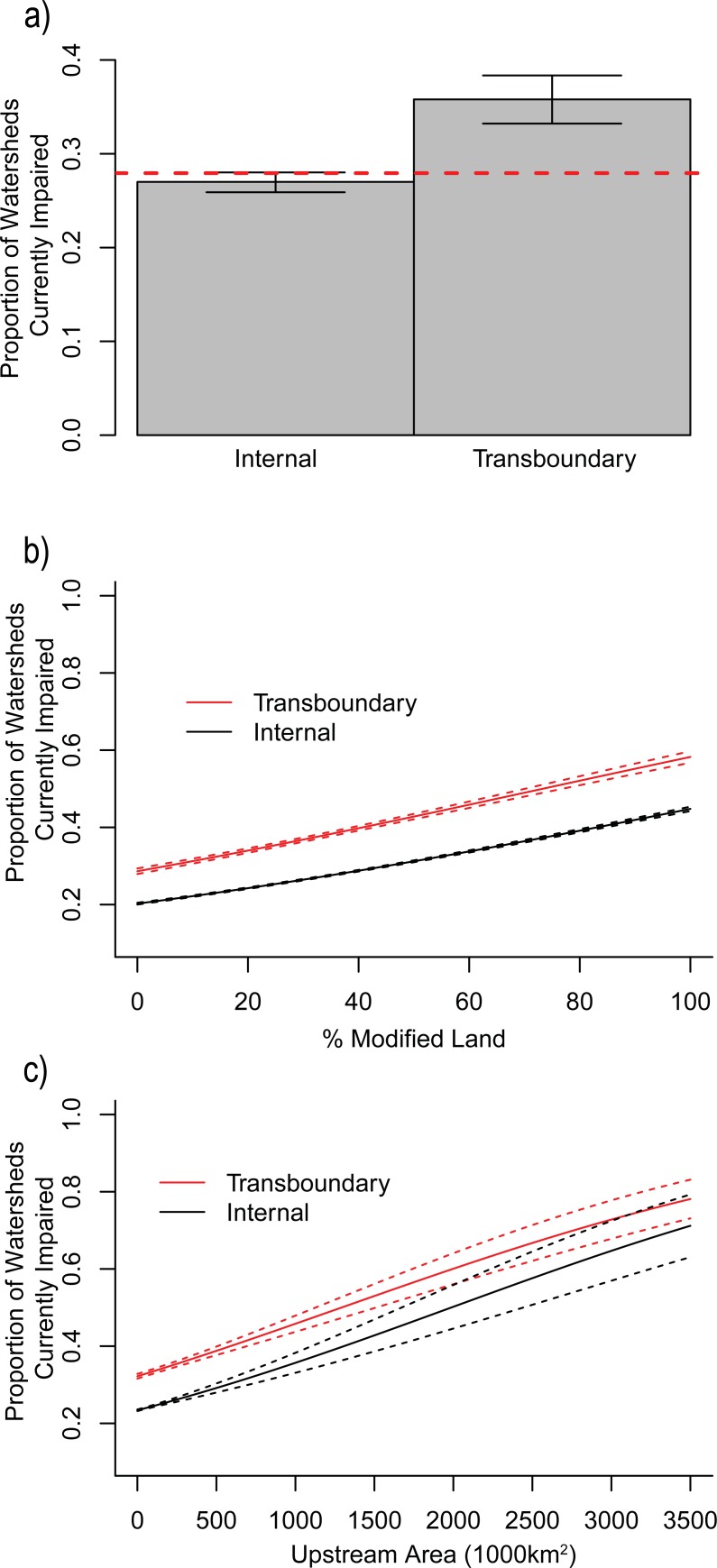
Factors associated with chances of watershed impairment. (a) Null modeling results where transboundary watersheds are more likely to be impaired than internal watersheds. (b) Logistic regression results demonstrating that watershed impairment increases as human land modification increases. (c) Logistic regression results demonstrating that watershed impairment increases as upstream area increases.

**Fig 4:**
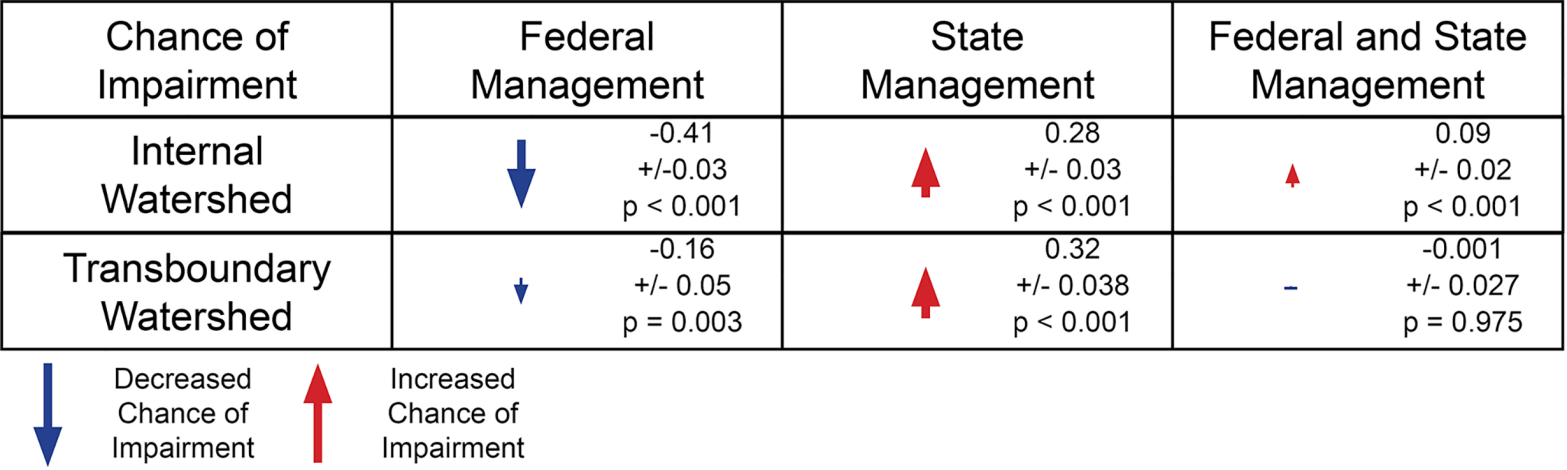
Chance of impairment for internal and transboundary watersheds based on Federal or State management jurisdiction. The presence of an arrow in the table indicates a statistically significant result. The direction and size of each arrow represents the direction (positive or negative) and relative magnitude of the management category’s effect on chance of impairment.

We aimed to understand the characteristics that are associated with impairment across United States watersheds. Specifically, we were interested in determining whether transboundary watersheds were more likely to be impaired than internal watersheds, and if so, what were the potential mechanisms driving these differences. We combined null modeling and logistic regressions to assess how geographical (upstream catchment area, modified land cover), and sociopolitical attributes (jurisdictional count) are related to a watershed’s likelihood of impairment. We found that transboundary watersheds were more likely to be impaired than internal watersheds (χ^2^_(2, N = 60,726)_ = 232.83, p < 0.001 Fig 3A). Although upstream catchment area and modified land cover impacted the likelihood of watershed impairment, they did not account for the differences observed between transboundary and internal watershed impairment (Fig 3B and 3C). Instead, we found a strong relationship between the number of land-owning agencies, transboundary or internal watershed groups, and impairment status (Fig 4).

Overall, there were 24.7% more impaired transboundary watersheds than the expected proportion in the null model. In contrast, the proportion of impaired internal watersheds was not significantly different than what was expected in the null model (Fig 3A). While these results provided support for our hypothesis that transboundary watersheds are proportionally more impaired than internal watersheds, they alone did not uncover any mechanisms associated with watershed impairment. Thus, we further investigated how upstream catchment area and modified land cover were affecting impairment likelihood within each watershed group. We hypothesized that transboundary watersheds would respond more severely to these attributes due to compounding socio-political effects.

Logistic regressions revealed that while impaired watersheds were associated with increased land cover and upstream catchment area, watershed transboundary status was independently associated with higher likelihood of impairment (Fig 3B and 3C). We found that for both transboundary and internal watersheds, the likelihood of impairment increased with greater modified land cover (p < 0.001) and upstream catchment area (p = 0.047). We observed an interaction between these two variables, where watersheds with larger upstream area were more likely to have high levels of modification (p = 0.016). Also, above upstream catchment areas of 2,000,000 km^2^, transboundary and internal watersheds did not have significantly different proportions of impairment, as the confidence intervals of both groups began to overlap. Importantly, however, these geographical attributes did not drive the observed differences in impairment probabilities between transboundary and internal watersheds. The transboundary or internal designations of watersheds did not affect the severity of impairment from modified land cover or upstream catchment area (p = 0.866 for transboundary, p = 0.804 for internal). Unpaired t-tests showed that neither upstream catchment area (p = 0.80) nor percentage of modified land (p = 0.87) significantly differed between transboundary and internal watersheds. Our results indicated that while the geographical attributes of watersheds strongly influence water quality, they were not driving the differences between transboundary and internal watershed impairment.

Our results did reveal significant relationships between the number of state and federal agencies operating within a watershed and the likelihood of impairment. Crucially, the relationships between our jurisdictional count and likelihood of impairment varied between transboundary and internal watersheds. When we first compared watersheds that contained public lands to watersheds that were entirely private, we observed that watersheds containing public lands were overall less likely to be impaired than private watersheds (p < 0.001). This suggested that, at the broadest level (i.e. public versus private), watersheds that are dominated by land belonging to non-governmental entities can be expected to have higher levels of impairment. Next, within the subset of watersheds containing public land, we investigated whether the number of state and federal agencies managing land in the watershed was associated with impairment. Here, we found that probability of impairment was associated with an interaction between the number of management agencies and the transboundary or internal designation of a watershed (p < 0.001). To understand this interaction, we split the dataset once more into two groups: transboundary and internal watersheds. For each group, we analyzed the relationship between the number of state and federal agencies and the likelihood of impairment. For both internal and transboundary watersheds, increases in the number of federal agencies were associated with a reduction in impairment likelihood, while increases in the number of state agencies were associated with increased impairment likelihood. Although having a higher number of state agencies was associated with a higher likelihood of impairment for both watershed groups, the negative effects of state agencies on watershed impairment were magnified in transboundary watersheds (Fig 4). Contrastingly, the positive effects of federal agencies on watershed impairment were higher in internal watersheds. Overall, our results provide evidence that: (1) transboundary watersheds are more likely to be impaired, and (2) jurisdictional fragmentation is associated with watershed impairment.

Even though state and national borders are often arbitrarily drawn, they may nonetheless have tangible impacts on the impairment probability of water bodies. For example, boundaries may incentivize polluting by externalizing the consequences of pollution to downstream jurisdictions, whether they be countries, states, or oceans. Transboundary spillover effects have been found to occur with industrial facilities in border counties within the United States[28]. Researchers have also identified higher levels of polluting activity upstream of borders between European nations[52], Brazilian counties[53], and Chinese provinces[54]. While we may intuitively expect higher potential for spillover effects between countries with non-overlapping legal and bureaucratic frameworks, the above examples demonstrate that subunits of a single country are no less immune to this phenomenon. Moreover, spillover effects may occur in direct response to federal decisions. In the case of China, a 2001 pollution reduction mandate issued by the central government loosened pollution enforcement and increased concentrations of polluting facilities just upstream of provincial borders[54]. We suspect that transboundary spillover effects may be contributing to our impairment results, especially given no significant differences in modified land cover or upstream catchment area between transboundary and internal watersheds.

The magnified effect of the jurisdictional count on impairment for transboundary watersheds is particularly compelling. It suggests that effective water resource management may be hindered by the presence of higher numbers of agencies, and that the existence of a border or coastline may compound this difficulty. One possible explanation for this result is based on “the diffusion of responsibility”[55]. This term originates from the field of sociology and refers to the phenomenon of individuals feeling diminished responsibility for actions as group size increases. The diffusion of responsibility has been shown to inhibit individual and collective actions across many contexts, from emergency interventions[55] to charitable donations[56] to corporate decision-making[57]. In our context, as more local agencies become involved in managing a watershed, the more difficult it may become for groups to implement Beneficial Management Practices for land use and water resources. While the diffusion of responsibility may help explain the observed correlation between the number of state agencies and watershed impairment in general, the situations may be different in transboundary watersheds. Agencies upstream of the border may feel less inclined to intervene when water pollution is transported out of their jurisdictions, while agencies downstream of the border may feel diminished responsibility if water pollution is entering from outside their jurisdictions. Conversely, another possible explanation connecting impairment and the jurisdictional count may be multiple agencies establishing themselves within a watershed in order to address severe waterbody impairment. However, as our results are correlative, we cannot distinguish between these possibilities. The opposite effects were seen when considering how the number of federal agencies impact watershed impairment. We suspect that since many federal lands are restricted-use (e.g. national parks, wilderness areas), they are likely to have a cumulative positive impact, as opposed to state lands that are often mixed-use and open to natural resources extraction.

It should be noted that higher numbers of agencies could simply mean that there are ‘too many seats at the table’ to allow for efficient decision-making at the collective level. Thus, the problem may not be that each agency feels less inclined to act, but rather that each agency has its own goals and vision for addressing a given managerial concern, creating gridlock within the collective. This has been identified as a challenge in multi-agency settings such as the Colorado River[27], the Israeli water sector[58] and urban watersheds in Canada[59]. At smaller scales, one solution may be found in overarching watershed partnerships that promote interagency coordination and public participation, thereby avoiding ‘silo effects’ between agencies or stakeholder groups[14,59,60]. Regardless of the mechanisms involved, our results imply that jurisdictional fragmentation may be a strong determinant of watershed impairment.

### Limitations and recommendations

Though the dataset we analyzed was nationwide and comprehensive, there are several potential limitations associated with its use. First, the EPA dataset of 303(d) impaired waters may be susceptible to interstate differences in water quality reporting. Under the Clean Water Act, states establish their own Total Maximum Daily Load programs so that their waterbodies may be suitable for designated “beneficial uses”[61]. The beneficial use of a waterbody may determine the water quality indicators that its managers are most interested in, thus allowing room for subjective variation when reporting impairment. Additionally, our analyses were constrained to waterbodies that were impaired in 2017. Since we did not use time series data, we were unable to assess whether watershed impairment trends were due to legacies of land uses such as mining and grazing.

Despite these limitations, we believe that the breadth of the dataset and the strength of our results highlight potential issues associated with transboundary watersheds. We recommend that future investigations incorporate nationwide datasets on point source pollutants, water abstraction and impoundments in United States waterbodies. These mechanisms of watershed impairment are not inherently connected with land modification and thus were not captured in our analyses. Such investigations may reveal the roles that transboundary spillover effects and overexploitation have in driving the observed differences between transboundary and internal watershed impairment. Additionally, time series analyses of watershed impairment and case studies of jurisdictionally fragmented watersheds could provide historical and local perspectives that were absent from this study.

We have provided evidence that transboundary watersheds are hotspots of impairment and that jurisdictional fragmentation is likely contributing this impairment. We are not proposing a one-size-fits-all managerial solution, nor claiming that all transboundary watersheds are subject to the same stressors. Rather, we recommend that watershed managers should assess the influence of jurisdictional fragmentation on a case-by-case basis. Our results also highlight the importance of considering Integrated Watershed Management policies as potential solutions to issues of water quality in jurisdictionally fragmented watersheds. Implementing boundary-spanning frameworks for group decision-making and non-point source abatement may often prove to be difficult. Fortunately, case studies such as Chesapeake Bay Watershed Agreement[62,63], watershed governance of Lake Tahoe [64], and the international management of Lake Constance[65], can provide insight into the shared characteristics of successful watershed management programs. While much is context-dependent, policies that expand public participation and streamline information sharing among agencies have been identified as crucial for properly balancing human development and watershed protection[14]. Given an ever-increasing need for clean freshwater due to rising populations, increased drought severity and food insecurity, it will be essential to more fully comprehend how our own socio-political landscapes impact the water resources we depend on.

## Supporting information

S1 Table Federal land management agencies listed in the Federal and Indian Lands datasets of the U.S. Geological Survey’s National Map program. Table includes the total approximate area of the lands for which each agency is designated as the primary administrator (within the contiguous 48 United States).
(DOCX)

S2 Table Broad categories of local owners of state lands, as designated in the Protected Areas Database of the United States. The “Other or Unknown” classification primarily consists of all the state lands of Minnesota, Iowa, and Illinois, as well as various lands including certain State Parks, Resource Management Areas, Conservation Areas, Marine Protected Areas, Conservation Easements Public Universities.
(DOCX)
