# Supplementary material for: Relationships between borders, management agencies, and the likelihood of watershed impairment
Source: PLoS One. 2018 Sep 20;13(9):e0204149. doi: 10.1371/journal.pone.0204149 (PMC6157817; doi:10.1371/journal.pone.0204149)
Supplement: S1 Table — Table includes the total approximate area of the lands for which each agency is designated as the primary administrator (within the contiguous 48 United States). (DOCX) [file pone.0204149.s001.docx]

| **Federal Administrative Agency [Primary]** | **Acronym** | **Area in the Contiguous 48 (square kilometers)** |
| --- | --- | --- |
| USDA Forest Service | FS | 839,270.81 |
| Bureau of Land Management | BLM | 702,316.77 |
| Bureau of Indian Affairs | BIA | 266,185.37 |
| National Parks Service | NPS | 109,998.80 |
| Department of Defense | DOD | 81,768.66 |
| Fish and Wildlife Service | FWS | 52,343.33 |
| Bureau of Reclamation | BOR | 9,786.60 |
| Department of Energy | DOE | 7,977.74 |
| Tennessee Valley Authority | TVA | 2,476.65 |
| Department of Agriculture | USDA | 808.57 |
| National Aeronautics and Space Administration | NASA | 586.13 |
| Department of Transportation | DOT | 131.51 |
| Metropolitan Washington Airports Authority | MWAA | 48.18 |
| Department of Veterans Affairs | VA | 42.83 |
| Department of Justice | DOJ | 23.14 |
| Department of Corrections | DOC | 10.18 |
| Department of Health and Human Services | HHS | 3.76 |
| Department of Labor | DOL | 3.35 |
| General Services Administration | GSA | 2.85 |

**S1 Table** Federal land management agencies listed in the Federal and Indian Lands datasets of the U.S. Geological Survey’s National Map program. Table includes the total approximate area of the lands for which each agency is designated as the primary administrator (within the contiguous 48 United States)
