# Supplementary material for: Relationships between borders, management agencies, and the likelihood of watershed impairment
Source: PLoS One. 2018 Sep 20;13(9):e0204149. doi: 10.1371/journal.pone.0204149 (PMC6157817; doi:10.1371/journal.pone.0204149)
Supplement: S2 Table — The “Other or Unknown” classification primarily consists of all the state lands of Minnesota, Iowa, and Illinois, as well as various lands including certain State Parks, Resource Management Areas, Conservation Areas, Marine Protected Areas, Conservation Easements Public Universities. (DOCX) [file pone.0204149.s002.docx]

| **Owner Name** | **Area in the Contiguous 48 (square kilometers)** |
| --- | --- |
| State Department of Natural Resources | 106154.96 |
| State Land Board | 105885.16 |
| State Department of Land | 66728.98 |
| State Fish and Wildlife | 56678.96 |
| Other or Unknown State Lands | 53047.67 |
| State Parks and Recreation | 19623.82 |
| State Department of Conservation | 13116.03 |
| Joint | 62.77 |
| Unknown | 30.17 |

**S2 Table**. Broad categories of local owners of state lands, as designated in the Protected Areas Database of the United States. The “Other or Unknown” classification primarily consists of all the state lands of Minnesota, Iowa, and Illinois, as well as various lands including certain State Parks, Resource Management Areas, Conservation Areas, Marine Protected Areas, Conservation Easements Public Universities
